# The Effects of Real-Time Haptic Feedback on Gait and Cognitive Load in Older Adults

**DOI:** 10.1109/TNSRE.2025.3578865

**Published:** 2025

**Authors:** Sharafian M. Ehsan, Colby Ellis, Ben Sidaway, Marie Hayes, Babak Hejrati

**Affiliations:** Department of Mechanical Engineering, The University of Maine, Orono, ME 04469 USA; Department of Mechanical Engineering, The University of Maine, Orono, ME 04469 USA; School of Physical Therapy, Husson University, Bangor, ME 04401 USA; Department of Psychology, The University of Maine, Orono, ME 04469 USA; Department of Mechanical Engineering, The University of Maine, Orono, ME 04469 USA

**Keywords:** Gait training, older adults, haptic feedback, cognitive load, stride length

## Abstract

Gait speed is a key indicator of mobility and health in older adults, with declines often reflecting neuromotor deficits rather than musculoskeletal or cardiopulmonary limitations. This study presents a wearable smartphone-based haptic feedback system that applies feedback to the thighs to increase peak thigh extension (PTE) and, consequently, improve stride length and walking speed. Thirty community-dwelling older adults (79.9 ± 6.5 years) participated in this study. Three treatment conditions were evaluated: (1) tactile feedback due to incorrect response when PTE was below the target ($F_{IR}$); (2) tactile feedback due to correct response when the target PTE was met ($F_{CR}$); and (3) verbal instructions ($I_{V}$) without feedback. Cognitive demand during treatment was assessed using a probe reaction time task. Walking trials comparing baseline with treatment conditions were conducted. We found significant differences for all gait parameters across walking trials (*p* < 0.001), but no significant difference among the three treatment conditions. The haptic feedback system significantly increased stride length by 14% and gait speed by 18%. Gains in speed and stride length were achieved using the haptic system during a single session, comparable to following verbal instructions. Although no statistical difference was found across treatments, thigh feedback employed a different mechanism than verbal instructions for attaining greater speed. Adding haptic feedback increased reaction time, but these increases were small ($F_{IR}$: 27ms, $F_{CR}$: 74ms), indicating minimal cognitive load. The observed gait improvements suggest haptic feedback is a viable option for gait training for older adults.

## I. Introduction

Many older adults demonstrate reduced gait speed without a concomitant decline in force production ability, cardiopulmonary restriction, or range of motion loss [1]. Such a reduction is instead attributed to a deficit in neuromotor control [2] as evidenced by decreased stride length [3] and increased variability in certain gait parameters [4], [5]. In such cases, interventions targeting the musculoskeletal and cardiopulmonary systems, such as strengthening, endurance, and flexibility exercises, have resulted in modest improvements in gait [1]. Instead, it is proposed that interventions should focus on affecting a change in the motor control system [4].

To improve gait speed, walkers must increase their stride length and/or stride rate (cadence) [6]. In older adults, stride length decreases with age, while cadence remains relatively unchanged or may increase slightly to maintain walking speed [7], [8]. Stride length has been suggested to be a more stable and modifiable parameter suitable for gait training [9]. To compensate for decreased stride length, Hollman et al. [10] reported that older adults exhibited increased cadence. Older adults with reduced stride length also experience higher energy costs when walking and often show an asymmetrical gait pattern [11]. It was reported by Eikema et al. [12] that manipulating stride length rather than cadence resulted in a more consistent stride length-to-velocity ratio essential for gait improvement. A longitudinal study showed that while both faster cadence and longer stride length were protective of speed decline, stride length had a strong association with sustained improvements [13]. Another study reported that older adults with frailty prioritize cadence as a compensatory mechanism to maintain speed when stride length diminishes; however, when compensatory capacity is exceeded, overall gait speed declines [14].

Wearable haptic feedback systems offer significant potential for individualized and home-based gait training thanks to their seamless integration into clothing, lightweight design, and advanced technology. Chen et al. [15] developed a custom-made ankle bracelet that provided feedback to retrain gait by matching foot progression angle and stride width. To reduce resultant tibial acceleration in runners, Sheerin et al. [16] investigated the effectiveness of real-time haptic feedback systems. Their findings indicated that haptic feedback was more efficient, less invasive, and less expensive than other modalities such as visual feedback systems. For gait balance training, Lee et al. [17] used a neck-mounted haptic device, demonstrating significant improvements in balance under specific task conditions.

Emphasizing the potential for home-based applications, our research group explored various haptic approaches, alongside other studies that have demonstrated haptic feedback systems used for modifying arm swing to enhance gait parameters such as speed in young and older adults, by improving kinematic parameters such as cadence and stride length [18], [19], [20].

In designing a training program to increase stride length in older adults, several kinematic and kinetic variables might be used for feedback. One such variable is the amount of thigh extension at the time of push-off in the trailing leg [21], [22]. The more the thigh is extended, the longer the contralateral step will be. Noghani et al. [23] showed that the peak thigh extension (PTE) at push-off was closely correlated with stride length and that haptic feedback during PTE could improve stride length and gait speed in young adults. Additionally, a previous pilot study from our group [24] in a small cohort of community-dwelling older adults used the same haptic feedback system and found enhanced stride length and gait speed compared to baseline performance. The haptic feedback system employed in this previous work was designed to be wearable and to give walkers customized information regarding their real-time performance over long periods. Other researchers have shown that feedback for rehabilitation [25], [26] can influence kinematics, force generation [27] and muscle activation patterns [28] during functional tasks [29], however, no studies to date have examined the potential benefit of haptic feedback on thigh extension and gait speed.

To use haptic feedback for gait training, more information is required to ascertain the optimal mode for providing such feedback. For example, should feedback be provided to the walker when a desired movement response is exhibited, or should the feedback be provided when the desired movement is not exhibited? Furthermore, if haptic feedback is to be incorporated into a walking-for-exercise regime, it is critical to determine the attentional load being placed upon the walker by this system and to track change over time with more training. Can older adults incorporate such feedback while still safely paying attention to the environment? Limited studies have explored the cognitive load imposed by feedback systems during gait training. Yasuda et al. [30] found that employing a digit subtraction task as a secondary task imposed a low cognitive cost while using a haptic feedback system to enhance postural control in older adults. Baer et al. [31] showed that visual biofeedback increased cognitive load significantly for younger adults, while Shull et al. [32] reported significant cognitive demands due to the use of haptic feedback to change foot progression angle during gait. With the long-term goal of enabling home-based gait training for older adults, the current study presents a haptic feedback system with two modes of presenting haptic feedback, one signaling that an incorrect response was made, i.e., the thigh was not extended enough, while the correct condition provided feedback when the thigh angle was sufficiently extended. This study also assessed the cognitive demand of the two feedback modes, measured using a probe reaction time task, and compared it to the cognitive load experienced by older adults when given verbal instructions to increase thigh extension before walking.

## II. Methods

### A. Haptic Feedback System

**Fig. 1.**
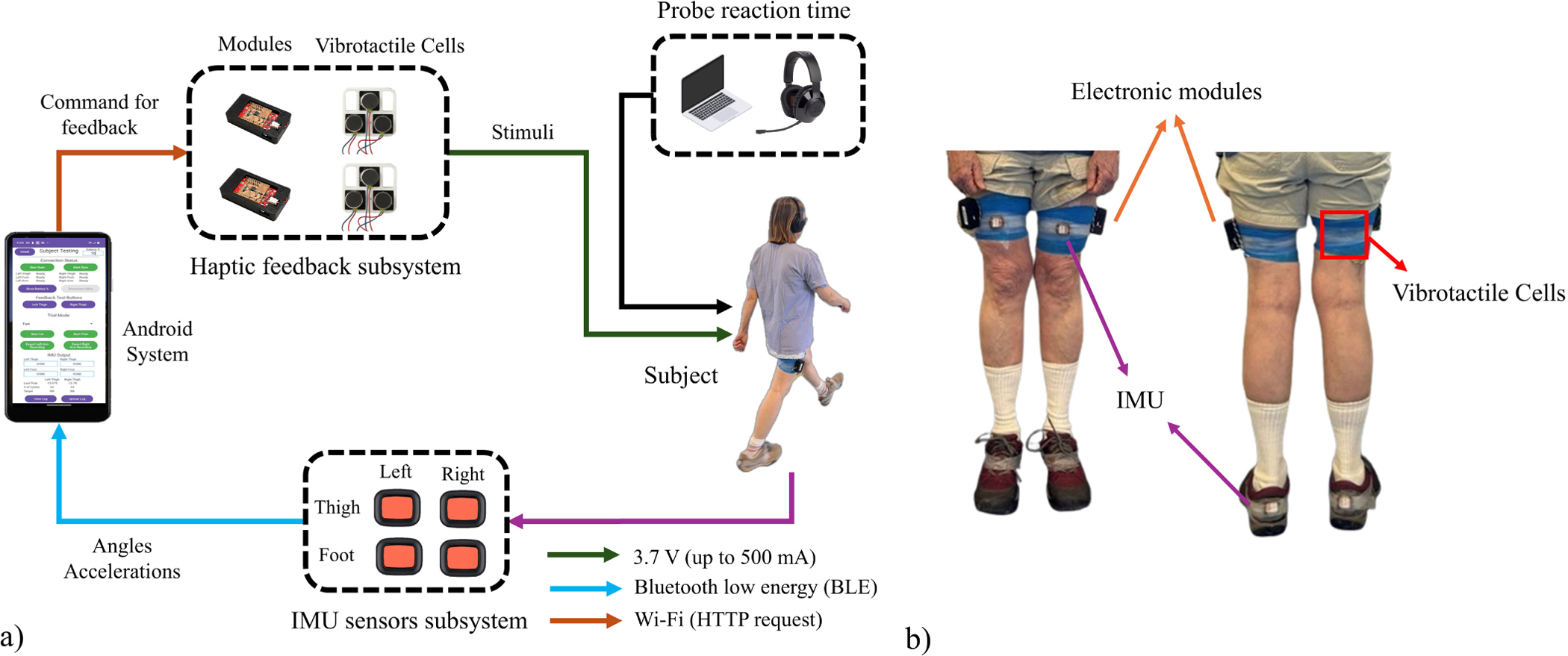
a) Diagram of the haptic feedback system with a participant in the loop and b) a participant wearing the system.

Fig. 1(a) shows the schematic of the haptic feedback system comprised of four IMUs (Xsens Technologies B.V., Enschede, The Netherlands), two custom haptic units, each containing an electronic module and three vibration cells (each cell is 12mm, 3.6mm height, 75mA at 3.7V), along with a smartphone that controlled the entire system based on our group’s previous system [19], [23]. The developed system is low-profile and lightweight, with a total added mass to each leg of about 91 g. As shown in Fig. 1(b), two IMUs were placed on the anterior surface of the participant’s thighs, two IMUs were attached to the back of the participant’s shoes, and the vibrotactile (VT) cells were secured on the posterior surface of the thighs. For the controller unit, the algorithms were implemented on a Pixel 6a smartphone (Google, Mountain View, CA, USA) by an application developed by our group in Android Studio. The orientation and acceleration data from IMUs were streamed to the application at 60 Hz via Bluetooth Low Energy (BLE) 5.2 to evaluate the PTE in real-time, and the commands to the haptic system were sent as HTTP requests to the electronic modules. ESP8266 microcontrollers were connected to the phone’s WiFi network to activate/deactivate the vibration cells. Custom circuit boards caused the cells to vibrate with a frequency of 240Hz close to the 245Hz frequency of the maximum sensitivity of the Pacinian mechanoreceptors of human skin [23]. All the components are in a 3D-printed case with the size of 51.1mm × 33.4mm × 8.7mm, and weight of 34 g. A program was developed in MATLAB/Simulink (MathWorks Inc. 2024) to generate auditory signals in a wireless headset and then record the participant’s verbal response via a microphone (Fig. 1(a)) for evaluation of attentional demand during experimental trials.

### B. Feedback Algorithm

**Fig. 2.**
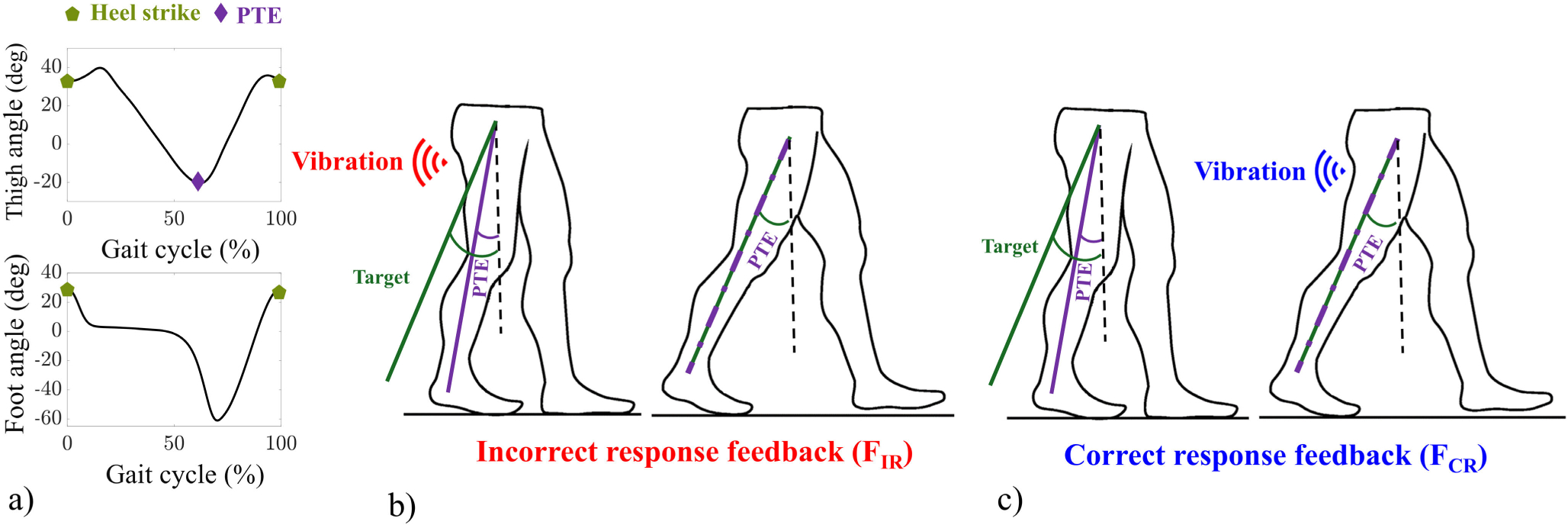
a) The thigh angle, the PTE, and foot angle during a gait cycle, b) the PTE and target angles during the incorrect response feedback mechanism in which the participants were signaled to extend their thigh to reach the determined target, and c) the same angles during correct response feedback where they received feedback to signal their success in attaining the target.

Two modes of haptic feedback were developed and implemented on a custom Android application; feedback confirming when a correct response (CR) was made ($F_{CR}$) and feedback indicating when an incorrect response (IR) was made ($F_{\text{IR}}$). Fig. 2(a) shows representative thigh and foot angles from which the participants’ PTEs and heel strikes were determined in real-time by the developed application and then used by the system for generating feedback. The determination of the individualized target PTE ($PT\;E_{\text{Target}}$) was performed automatically with the algorithm deployed in the developed application. As shown in Algorithm 1, each participant’s thigh angles were evaluated using the IMUs’ readings during baseline and fast walking trials and then used to automatically determine the PTEs by the application. The participant’s average PTEs during baseline ($PT\;E_{\text{Baseline}}$) and fast ($PT\;E_{\text{Fast}}$) trials were used by the application to calculate an individualized $PT\;E_{\text{Target}}$ [24]. To allow the participants to gradually adapt to the feedback and their $PT\;E_{\text{Target}}$, we first added half of the difference between $PT\;E_{\text{Baseline}}$ and fast $PT\;E_{\text{Fast}}$ as the increment to $PT\;E_{\text{Baseline}}$ (see lines 6–10 in Algorithm 1) to establish an initial target.

**Algorithm 1:** Initial Target Creating

**Input:** Streaming IMU data	
**Output:** $PT\;E_{\text{Target}}$	
1:	Streaming IMUs data including Euler angles and accelerations at 60 Hz during baseline and fast trial
2:	Evaluating thigh angles in the sagittal plane
3:	PTE← Find peak thigh extension from thigh angle
4:	$PT\;E_{\text{Baseline}}\;\leftarrow$ Average of last 40 PTEs of baseline trial
5:	$PT\;E_{\text{Fast}}\;\leftarrow$ Average of last 40 PTEs of fast trial
6:	$Increment\leftarrow \left(PT\;E_{\text{Fast}}\;-PT\;E_{\text{Baseline}}\right)/2$
7:	**if** Increment←2°, as two times of IMU dynamic error (1°) **then**
8:	Increment←2°
9:	**end if**
10:	$PT\;E_{\text{Target}\;}\leftarrow PT\;E_{\text{Baseline}\;}+\;Increment$
11:	**return** $PT\;E_{\text{Target}}$

During the first 10 strides in the treatment trials, the participants did not receive feedback so that they could reach comfortable steady-state walking [24]. After the 10^*th*^ stride, the haptic system started comparing a participant’s PTE with their initial target. Similar to the approach by Chen et al. [15], if the participant’s PTE was above the initial target in at least 80% of strides over the next 20 strides, then the initial target increased to their $PT\;E_{\text{Fast}}$ for both feedback groups (see Algorithm 2).

Both haptic feedback modes used the same algorithm for target determination. However, the feedback was provided differently, as shown in Algorithm 2. The goal of training with the feedback algorithms was to increase the participant’s gait speed by increasing stride length, either using verbal instructions, as a physical therapist might do or by providing haptic feedback designed to increase the PTE. The details of the two haptic feedback and verbal instruction conditions, $I_{V}$, are as follows:

**Algorithm 2:** Target Increase Determination and Treatment Conditions

**Input:** Streaming IMU data in treatment trial	
1:	**if** Number of strides since the start of treatment trial ≤ 20 **then**
2:	**exit**
3:	**end if**
4:	**if** $PT\;E_{\text{Target}}$ has previously increased **then**
5:	**continue**
6:	**else if** $|PTE|>\left|PT\;E_{\text{Target}}\right|$ over at least 80% the last 20 strides **then**
7:	$PT\;E_{\text{Target}\;}\leftarrow PT\;E_{\text{Target}\;}+Increment$
8:	**end if**
9:	**switch** Treatment condition
10:	**Case** Incorrect response ($F_{\text{IR}}$)
11:	**if** $|PTE|<\left|PT\;E_{\text{Target}}\right|$
12:	Send HTTP request for giving feedback
13:	**end if**
14:	**Case** Correct response ($F_{CR}$)
15:	**if** $|PTE|>\left|PT\;E_{\text{Target}}\right|$
16:	Send HTTP request for giving feedback
17:	**end if**
18:	**Case** Verbal Instruction ($I_{V}$)
19:	**exit**
20:	**end switch**

- $F_{\text{IR}}$ condition: The feedback algorithm signaled the participants to increase their PTE to the calculated target each time the PTE was below the target ($|PTE|<\left.\left|PT\;E_{\text{Target}}\right|\right)$ during the terminal stance phase of that leg [24], as shown in Fig. 2(b).
- $F_{CR}$ condition: The vibrocells were programmed to vibrate only when the PTE is above the target $(|PTE|>\left|PT\;E_{\text{Target}}\right|$), as shown in Fig. 2(c).
- $I_{V}$ condition: Before walking, participants were instructed to increase stride length by increasing thigh extension when walking.

Participants received feedback on every cycle of each leg when the PTE of that leg was below ($F_{IR}$) or above ($F_{CR}$) the target, depending on their assigned treatment condition. Providing feedback on every cycle (if needed) was similar to the method in previous studies using haptic feedback for gait training [15], [32].

The developed graphical user interface with an Android system enabled the researchers to switch seamlessly between different trials and conditions. After completion of all the experimental trials, all the data were automatically saved and uploaded as a CSV and a Log file to the cloud. After uploading, analysis was performed, as detailed in the Data Processing section.

### C. Participants

The study was approved by the University Institutional Review Board at the University of Maine (IRB 2019–04-15). All participants signed a written consent form before starting the study. Community-dwelling older adults aged 65 years or older (*N* = 30, age: 72.9 ± 6.5 years (range: 66 – 94), body mass: 72.31 ± 13.64 kg, height: 169.69 ± 8.74 cm, sex: 18 F/12 M) were recruited through flyers and advertisements at local community centers. The inclusion criteria required that participants could walk independently and continuously without using an assistive device for at least 10 minutes and converse in English. The exclusion criteria included a diagnosis of peripheral neuropathy or other neuromuscular diseases (e.g., Parkinson’s disease, multiple sclerosis, stroke), diagnosed cognitive impairment, or cardiopulmonary disease. The sample size of 30 was calculated using G*Power 3.1.9.4 based on a significance threshold of α=0.05,80% power, and a speed threshold of 0.1 *m/s* as a substantial meaningful change of speed [33], [34]. The 30 older adults were quasi-randomly assigned to one of three experimental treatment conditions (10 participants in each group). In the group assignment, we sought to maintain a similar average age and sex ratio across the groups.

### D. Experimental Design

**Fig. 3.**
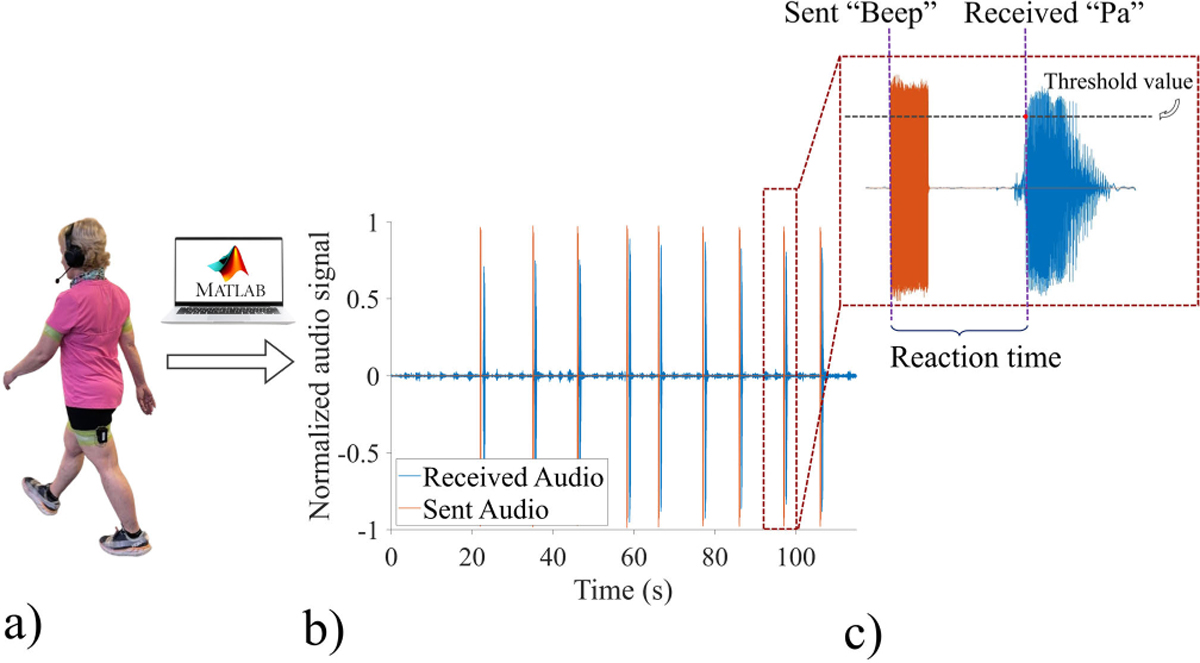
(a) A participant wearing headphones and a microphone during a feedback trial with a probe reaction time system. (b) The experimental setup used MATLAB/Simulink to send “Beep” auditory cues and record the participant’s “Pa” audio responses. (c) The reaction time is calculated by measuring the time difference between the sent cue and the corresponding response when the audio signals reach a fixed threshold value.

The first aim of this experiment was to compare the two feedback methods and investigate whether haptic feedback can change walking patterns similar to when verbal instructions are given. A secondary aim of the study was to examine whether the haptic feedback system would impose any significant attentional load, as indexed by probe reaction time, during walking. In all conditions, a probe reaction time task was added to two of the gait trials, during which participants responded to auditory cues presented in headphones (Fig. 3(a)). In each trial involving the probe reaction time task, a sequence of nine auditory cues of “beep” with variable times between the cues was presented to the participants who would respond “Pa” as quickly as possible when the target was heard [35] (Fig. 3(b)). The participants’ audio responses were recorded and amplitude normalized using a custom Simulink program. The reaction time was calculated as the time difference between the onset of an auditory cue and the participant’s response received as an audio signal at a predefined threshold (Fig. 3(c)). This threshold was experimentally determined to distinguish audio responses from the environmental audio noise. For consistency, the same fixed threshold value was applied to all participants across all the trials with the probe reaction time system. The time interval between the cue and the corresponding response was averaged over the last eight cues, serving as an index of cognitive demand in that trial for a participant.

**Fig. 4.**
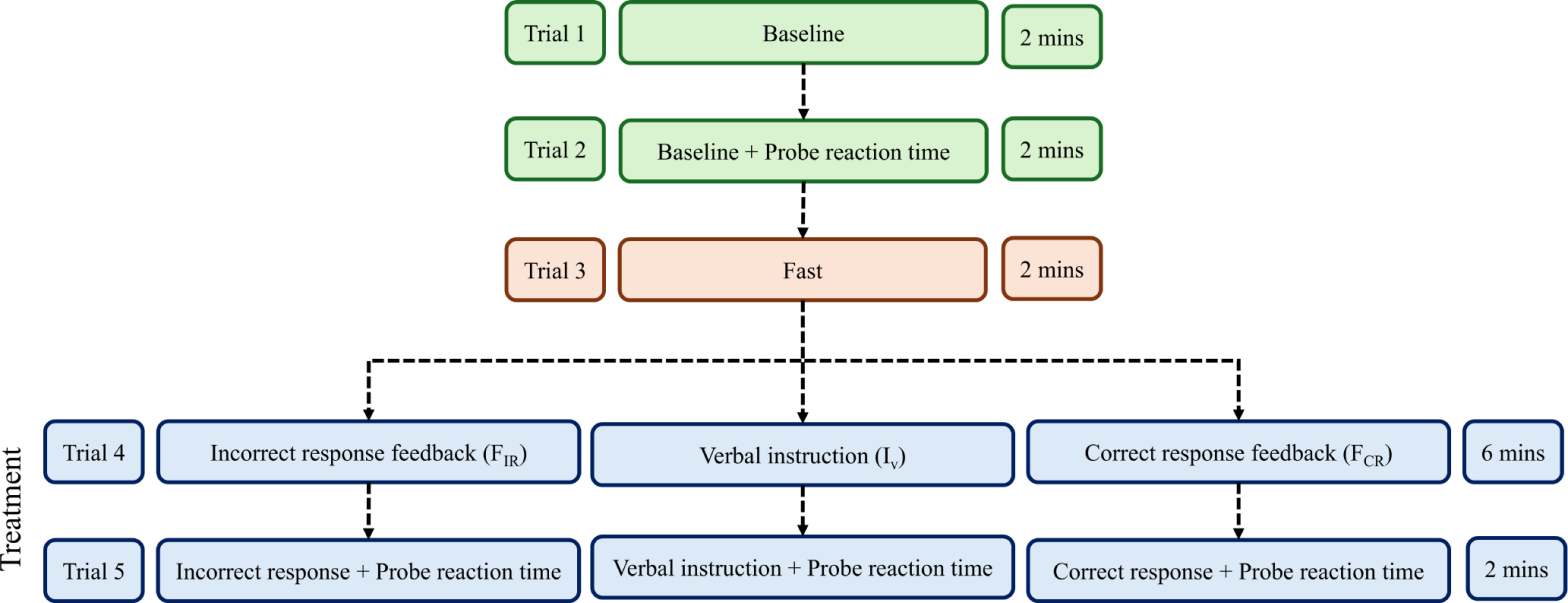
The sequence of the experimental trials detailing the progression from the baseline to treatment trials.

The experiments were conducted on an indoor oval athletic track, a standard 200-meter track that has gentle curves with a radius of approximately 19 meters. Participants were instructed to walk on the innermost lane, which provided a flat surface and smooth transitions through the curves. Five walking trials were conducted on the track. The sequence of the trials and their time lengths are shown in Fig. 4. During trial 1 (two minutes), participants walked at their self-selected speed to establish an average baseline PTE to be used in the feedback-generating algorithm and to establish a baseline for comparison to the haptic feedback trials. In trial 2 (two minutes), participants walked at their self-selected pace but were required to verbally respond with “Pa” when an auditory beep cue was presented. In trial 3 (two minutes), the participants were asked to walk as fast as they safely could. These data were used for the feedback algorithm that would calculate the target PTE values for the feedback trials. In trial 4, the treatment trial, the participants walked in one of the three different treatment conditions: $F_{IR},F_{CR}$, and $I_{V}$ for six minutes. In trial 5, participants walked again in the same treatment conditions for 2 minutes while attentional load was assessed with the probe reaction time task.

Before starting Trial 4 (i.e., the treatment trial), participants in all three groups were shown a diagram illustrating what constituted thigh extension and how to achieve it. For the feedback groups ($F_{IR}$ and $F_{CR}$), participants then experienced the vibrational feedback on the posterior side of their thighs to become familiar with the sensation. Following the same procedure as in previous studies [23], [24], participants in the $F_{IR}$ group were shown a figure similar to Fig. 2(b) and were instructed that when they felt vibration on their thighs, they should further extend their thighs in the next cycle. If they had already achieved sufficient thigh extension, they would not receive any vibration. Thus, the goal was to avoid feeling vibrational feedback. For the $F_{CR}$ group, a figure similar to Fig. 2(c) was used to instruct them that feeling vibration confirmed adequate thigh extension and correct movement. Therefore, the goal was to receive vibrational feedback. No instructions regarding walking speed were given to any of the participants. For the $I_{V}$ group, participants were asked to lengthen their steps by increasing thigh extension during walking. After the instructions, all participants in the three groups completed a short walking bout to become familiar with their assigned training method and to have the opportunity to ask any questions.

Following the completion of the trials, clinical and cognitive tests were conducted. These included the Montreal Cognitive Assessment (MoCA) [36] for cognitive assessment and the Functional Gait Assessment (FGA) [37], the Timed Up and Go test (TUG) [38], and the Activities-specific Balance Confidence (ABC) [39] scale for gait assessment. Finally, the participants were asked to evaluate the haptic feedback system on a scale of 1–10 by answering specific questions about a) comfort while walking with the feedback, b) promptness of receiving feedback, c) the noticeability of vibrations during feedback, and d) the intuitiveness of adjusting gait to the feedback.

### E. Data Processing

We used the last 40 gait cycles during each trial for evaluating the biomechanical parameters. Stride length, defined as the distance between two consecutive heel strikes of the same foot, was determined by integrating the acceleration data and applying the zero-velocity update method [24], [40] under the assumption that foot velocity is zero during flat-foot contact. Cadence was determined by using the time of each heel strike, while speed for each gait cycle was calculated by dividing the stride length by its corresponding gait cycle time. To evaluate cognitive demand, the average participant’s reaction time to eight stimulus-response pairs during baseline and treatment trials with a probe (trials 2 and 5, respectively) was compared. The percent change for each gait parameter was calculated between the baseline and treatment values to demonstrate changes in the parameters of interest relative to their baseline values.

### F. Statistical Analysis

Statistical analyses were conducted using SPSS v29 (SPSS Inc., Chicago, IL, USA). Univariate ANOVAs were used to compare age, BMI, MoCA, FGA, ABC, and TUG across treatment conditions. Also, a mixed-model ANOVA with a significance level of α=0.05 was used, in which the walking trial (i.e., baseline, fast, and treatment trials) was the within-subjects factor and the treatment condition ($F_{IR},F_{CR},I_{V}$) was the between-subjects factor. The dependent variables in the analyses were PTE, stride length, cadence, gait speed, and reaction time. To compare gait during the trials with the probe reaction time task (trials 2 and 5) to the baseline and treatment trials without the task (trials 1 and 4), we used a three-way mixed-model ANOVA with walking trial (baseline, treatment) and probe status (absent, present) as within-subjects factors with treatment condition ($F_{IR},F_{CR},I_{V}$) as a between-subjects factor. Mauchly’s test was used to assess the sphericity assumption, and if violated, the Greenhouse-Geisser correction was applied. Post-hoc analysis with Bonferroni correction was performed to identify pairs of conditions with statistically significant differences.

## III. Results

### A. Participants

Table I shows the participants’ gait, cognitive assessments, and physical attributes. Analyses revealed no significant group differences in these parameters. The gender ratio and age were similar across the groups.

### B. Gait Outcomes

**Fig. 5.**
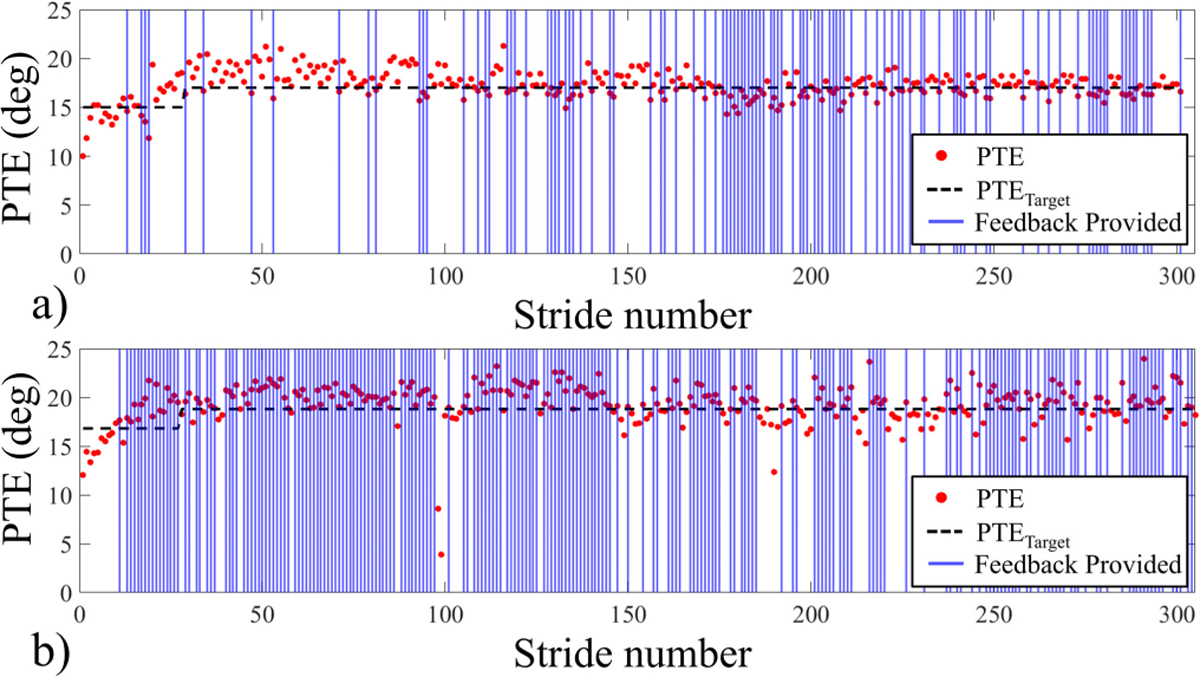
Examples of the provided feedback during treatment trials: (a) Incorrect response feedback ($F_{IR}$) was provided when PTE fell below the target with the target increasing once criteria from Algorithm 2 were met and (b) correct response feedback ($F_{CR}$) was given when PTE reached the target. After the initial target increased, it remained constant for the rest of the trial.

Fig. 5 shows examples of how haptic feedback was applied during each feedback method. Figure 5a demonstrates how feedback was sent to encourage the participant to reach the target whenever the PTE fell below the $PT\;E_{\text{Target}}$ during $F_{\text{IR}\;}$. The example also demonstrates how the target increased according to the rules outlined in Algorithm 2. The participant successfully met the criteria for increasing the target, and the target remained constant for the remainder of the trial. Fig 5b demonstrates similar information for $F_{CR}$ treatment condition.

**Fig. 6.**
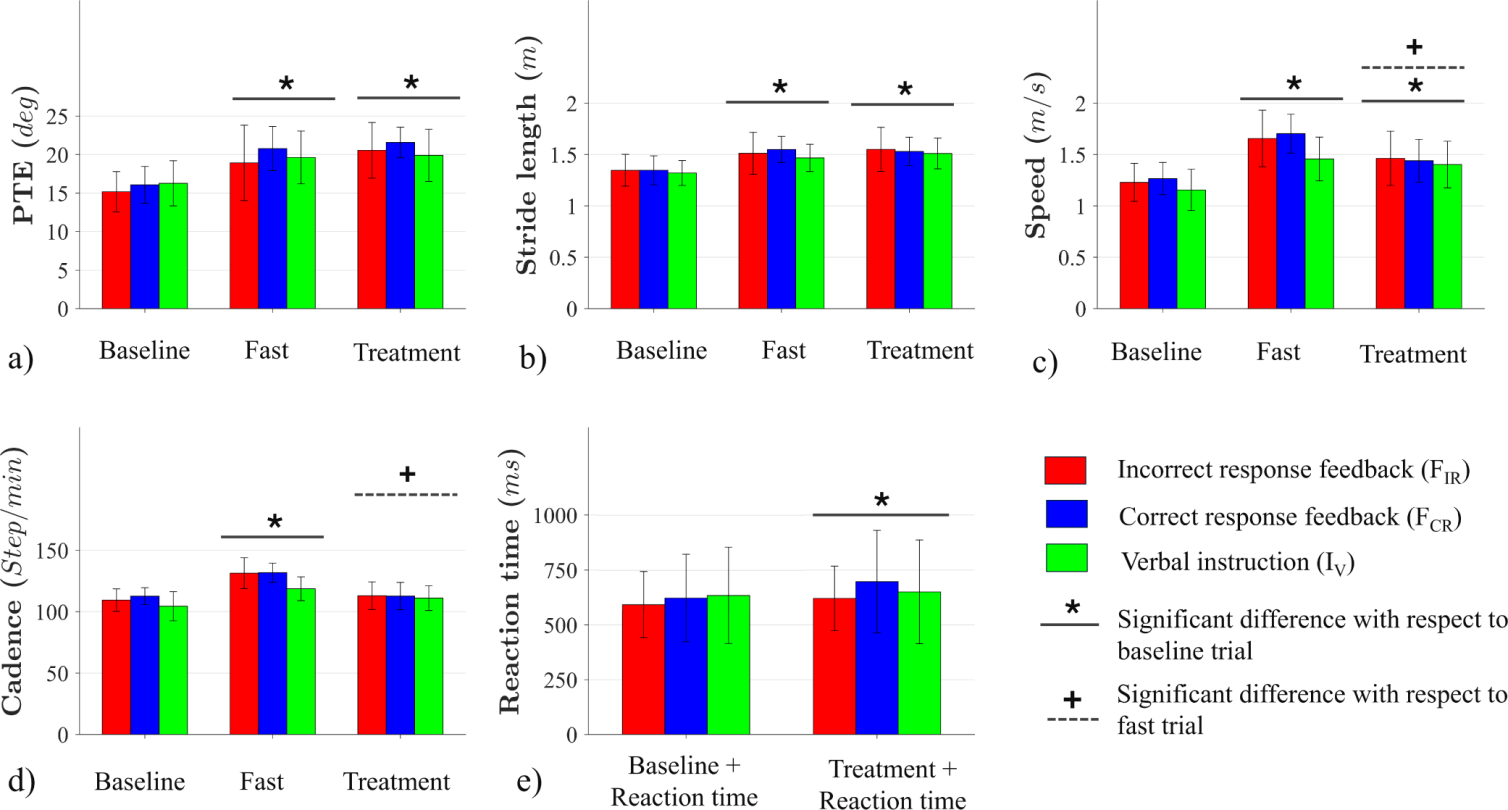
(a) PTE, (b) stride length, (c) speed, (d) cadence, and (e) reaction time for the three groups across the baseline, fast, and treatment trials with error bars showing standard deviations.

Table II presents the gait data as a function of treatment and trial, along with the percent change from the baseline to the treatment trial. As shown in Fig. 6a–d, the effect of the walking trial was significant for all the gait outcomes. Significant differences were found for the PTE (F(2,54)=68.72, *p* < 0.001, $\eta^{2}=0.72$), stride length (F(2,54)=95.75, *p* < 0.001, $\eta^{2}=0.83$), cadence (F(2,54)=68.12, *p* < 0.001, $\eta^{2}=0.71$), and speed (F(2,54)=87.09, *p* < 0.001, $\eta^{2}=0.76$) across the three baseline, fast, and treatment trials. Post-hoc analyses of PTE and stride length revealed significant increases compared to baseline in the baseline-fast ($p^{\prime }s\;<\;0.001$) and baseline-treatment ($p^{\prime }s\;<\;0.001$) comparisons. No significant difference was found for the fast-treatment comparison. For cadence, significant increases were found in the baseline-fast (*p* < 0.001) and fast-treatment (*p* < 0.001) comparisons, while no significant difference was observed in the baseline-treatment comparison. For speed, all pairwise comparisons of baseline-fast, baseline-treatment, and fast-treatment were significantly different ($p^{\prime }s\;<\;0.001$). Statistical analysis revealed no significant difference in PTE, stride length, cadence, and speed as a function of treatment condition ($F_{IR},F_{CR},I_{V}$). The interaction between the baseline trial and treatment condition was only significant for cadence (F(4,54)=2.61, *p* = 0.045, $\eta^{2}\;=\;0.16$), showing differences in cadence for all treatment conditions. In the $I^{V}$ condition, the baseline cadence was lower than in the fast and treatment trials ($p^{\prime }s\;<\;0.001$), while fast and treatment trials had a similar cadence. In the $F_{IR}$ and $F_{CR}$ conditions, no difference was found between baseline and treatment trials; however, cadence in these trials was lower than in the fast condition (*p* < 0.001).

The average (standard deviation) number of strides at which the initial target increased for participants in the $F_{IR}$ and $F_{\text{CR}}$ groups was 27.50 (2.17) and 27.30 (1.16), respectively. An independent t-test revealed no statistically significant difference between the two feedback groups in the number of strides taken before the individualized targets increased.

### C. Cognitive Demand

A similar statistical analysis to that used for gait parameters was performed with reaction time as the dependent variable. The only difference was that the walking trial only included baseline and treatment (trials 2 and 5) since there was no probe task during the fast trial. As shown in Fig. 6e, reaction time significantly increased in the treatment compared to baseline trials (F(1,27)=9.48, *p* = 0.005, $\eta^{2}=0.26$); however, no differences were observed among the three groups.

Additionally, a three-way ANOVA (walking trial, probe status, and treatment conditions) on each gait parameter revealed significant differences in all the parameters between the two trials (baseline vs. treatment). There was no significant difference in the PTE due to probe status, while stride length (F(1,27)=16.44, *p* < 0.001, $\eta^{2}=0.37$), cadence (F(1,27)=28.56, *p* < 0.001, $\eta^{2}=0.51$), and speed (F(1,27)=31.97, *p* < 0.001, $\eta^{2}=0.54$) increased significantly when the probe was present. The average (standard deviation) of the gait parameters for the probe absent and present, respectively, are as follows (probe absent, probe present, change): for the PTE (18.26 *(*1.30*)*, 18.52 *(*3.05*)*, 1.4%), stride length (1.43 *(*0.15*)*, 1.45 *(*0.14*)*, 1.4%), cadence (110.5 *(*9.35*)*, 113.4 *(*8.42*)*, 2.6%), and speed (1.32 *(*0.19*)*, 1.37 *(*0.18*)*, 3.8%).

### D. Participants’ Rating

The questionnaire average ratings (a 1–10 scale) in the $F_{IR}$ and $F_{CR}$ conditions were a) comfort: 9.26 ± 1.63, b) instantaneous feeling of the vibrations: 9.11 ± 1.51, c) noticeability of vibration: 9.04 ± 2.05, and d) intuitive gait adjustment: 7.71 ± 2.54. Wilcoxon-Mann-Whitney tests for two independent groups revealed no significant differences between the two haptic feedback conditions.

## IV. Discussion

This work evaluated the feasibility of a novel haptic feedback system for gait training in older adults. Two haptic feedback modes were compared to verbal instructions in terms of their ability to affect gait speed. The three treatment approaches encouraged the participants to increase their PTE, thereby increasing stride length and gait speed. Previous studies of older adults have shown the importance of stride length in improving gait speed [6], [12], [41] while haptic feedback has been shown to improve performance in both younger [23] and older adults [19].

The variable of focus, PTE, increased significantly in both the treatment and fast trials of all three groups ($F_{IR},F_{CR},\;I_{V}$) compared to their baseline trials; however, no significant differences in PTE were observed between the treatment and fast trials in any of the groups. Similar findings were observed for stride length in the two haptic feedback groups ($F_{IR}$ and $F_{CR}$), as expected given the dependence of stride length on PTE. The interaction analysis found no change in cadence between baseline and feedback treatment trials in the feedback conditions ($F_{IR}$ and $F_{CR}$), indicating that the increase in gait speed with feedback was driven by the increase in PTE but not cadence. This is also evident in Table II, where the percent changes of cadence in the feedback conditions compared to their baselines were not statistically different from 0%. In contrast, in the verbal instruction condition ($I_{V}$), cadence was used to increase speed and its percent change was significantly greater than 0%. These findings highlight the power of haptic feedback to cause a change in the target variable (PTE). Importantly, during the fast-walking trial, all participants chose to increase cadence as the means to increase gait speed, rather than stride length. Therefore, simply asking older adults to walk faster will not lead to an increase in stride length. Also, it should be noted that the participants walked at their maximum comfortable speed, which was sustainable only for a short period.

Parameter-focused feedback, such as personalized PTE improvements with haptic feedback, presents a promising option for increasing stride length in older adults with age-related walking decline. Regular training with verbal instructions by a physical therapist (PT) may not be possible or practical for many older adults due to financial and transportation challenges. Given similar concurrent improvements in both the verbal instruction and haptic feedback groups, this highlights the feasibility of using the feedback system when verbal instruction is unavailable, thus enabling regular and independent home-based training. A more comprehensive comparison is still needed to compare feedback and verbal methods and the retention of potential improvements for each method through a multi-session training study.

In a recent pilot study by Miyazaki et al. [42], concurrent auditory feedback regarding ankle and hip angles did lead to a 2.2% increase in gait speed and a 7.4% improvement in stride length in older adults while the feedback was present. In contrast, the haptic feedback used in the current study generated 14.32%–18.90% increase in gait speed and 14.04%–15.04% improvement in stride length (see Table II). The widely cited work by Perera et al., [43] reported that a range of 0.08 *m/s* to 0.14 *m/s* increase in gait speed was a substantial change. Our results revealed a mean improvement of 0.24 *m/s* in the $F_{IR}$ group and 0.18 *m/s* in the $F_{CR}$ group, demonstrating that our system could deliver significant speed improvements for older adults. Our results for improving gait speed align with this study and those of previous preliminary study from Hossain et al. [24] in which the mean speed improvement was approximately 18% with a 33.8% improvement for the PTE, compared to 14.32%−18.90% for speed and 35.81%–3 6.23% for the PTE.

The two haptic feedback modes examined in the current study increased gait speed equivalent to that engendered by specific verbal instructions to alter gait. Furthermore, the interaction in the cadence analysis showed that unlike in the $I_{V}$ group, cadence did not change in the feedback conditions of $F_{IR}$ and $F_{CR}$ compared to baseline. The haptic feedback regarding thigh angle had the desired effect of causing the participants to walk faster by taking longer strides without any significant change in cadence, which is argued to be more sustainable based on the literature. The lack of a significant increase in cadence in the feedback conditions is similar to Miyazaki’s study [42] that reported a 5% decrease in cadence during training with auditory feedback. The participants were encouraged to use their stride length rather than their cadence to increase their speed, and the obtained results confirm that participants achieved this goal. Regarding the number of strides each participant took before their target was increased, the results indicate that the type of haptic feedback did not influence how quickly participants responded to feedback and adapted their gait to reach the increased target.

The probe reaction time results revealed that the addition of concurrent haptic feedback did impose a statistically significant cognitive demand on the participants ($F_{IR}$: 27ms, $F_{CR}$: 74ms). Although reaction time slightly increased during the 6-minute feedback trials, this is likely due to the participants still being in the early skill acquisition phase during a single-session training, in which attentional load is expected to be higher and automaticity has not yet developed. From a motor learning perspective, such increases are typical in the early skill acquisition phase and tend to diminish with practice as automaticity develops [44], [45]. It is therefore possible that with multi-session training, and as the skill becomes more automatic, the reaction times will converge to baseline levels. While the observed delay of 27–74ms could influence the voluntary response to perturbations during walking, the probe reaction time primarily reflects cortical processing of auditory cues. In contrast, rapid postural responses to perturbations are largely mediated by subcortical structures, particularly the cerebellum and brainstem motor pathways (e.g., spinocerebellar tracts and rubrospinal outputs) [46], [47], and occur at reflexive latencies (< 100ms) independent of cortical involvement. Further research is still needed to explore the impacts of multi-session training on reaction time and automaticity, as well as the impacts of the feedback system on reactive balance during walking. Importantly, the attentional load created by the haptic feedback did not adversely affect any of the gait parameters recorded.

Although a slight yet statistically significant increase in walking speed was observed during the probe task, the mean difference of approximately 0.049m/s (3.8%) was much smaller than the 0.18–0.25m/s (14.32%22.43%) increases due to the walking trial effect. In practical terms, this suggests that probe tasks had minimal impact on cadence, stride length, and speed. While the auditory stimuli used in this study were not designed to influence gait, it is possible that their presence indirectly exerted subtle effects. Divided attention tasks involving emotionally charged auditory stimuli have been shown to elicit compensatory improvements in gait performance in cognitively intact older adults [48]. Additionally, auditory cues have been reported to subconsciously influence motor patterns, such as walking [49].

The subjective ratings of the system indicated that the feedback system was comfortable and intuitive to use, as indicated by their high ratings of items (a-c) in the questionnaire; however, more training is still needed, as indicated by relatively lower ratings of item (d). Although no statistical difference was found between the ratings of $F_{IR}$ and $F_{CR}$, participants with $F_{CR}$ who achieved consistent success with the target thigh angle expressed less enthusiasm about continuously receiving tactile feedback.

## V. Conclusion

The current study presented a haptic feedback system capable of increasing older adults’ stride length and, consequently, gait speed during the six minutes it was employed. The increases in gait speed were equivalent to those seen when people were given explicit verbal instructions. The system appeared to impose a minimal cognitive load and was readily accepted by the walkers who used the system. Now that the effectiveness of haptic feedback in affecting behavioral change has been established, several avenues of future research are apparent. The current work showed the immediate effects of the feedback while it was being given. However, it remains to be seen if these effects are retained when the feedback is no longer present. The retention of behavioral change should be assessed at several time intervals following the withdrawal of the feedback. In the current study, the feedback was provided for only six minutes on one day. Future research should investigate the effectiveness of the haptic feedback system in driving behavioral changes after multiple sessions of use.

## Figures and Tables

**TABLE I:** Physical Characteristics and Gait and Cognitive Assessments’ Results

	Incorrect Responses Feedback ($F_{\mathrm{IR}}$)	Correct Responses Feedback ($F_{\mathrm{CR}}$)	Verbal Instruction ($I_{V}$)
Number of participants	10 (6F)	10 (6F)	10 (6F)
Age	71.50 (4.79)	72.90 (7.60)	74.30 (7.15)
Height (m)	1.66 (0.08)	1.68 (0.09)	1.69 (0.10)
Body Mass (Kg)	72.25 (12.98)	69.83 (9.48)	69.38 (8.56)
BMI (*Kg/m*^2^)	26.06 (5.52)	24.73 (3.22)	24.14 (3.02)
MoCA	25.90 (2.84)	26.40 (2.63)	23.90 (2.07)
TUG	8.37 (2.18)	7.54 (1.05)	8.56 (2.27)
FGA	23.80 (3.93)	24.00 (3.01)	22.40 (5.38)
ABC	89.05 (12.32)	95.65 (2.96)	91.46 (7.21)

**TABLE II:** Means (Standard Deviations) and Percent Changes of the Gait Parameters for the Baseline, Fast, and Treatment Trials (Trials 1, 3, and 4, Respectively) as Well as Reaction Time for the Baseline and Treatment Trials With Probe Reaction Time Task (Trials 2 and 5, Respectively) for Each Treatment Condition

	Incorrect response feedback ($F_{\mathrm{IR}}$)				Correct response feedback ($F_{\mathrm{CR}}$)				Verbal Instruction ($I_{V}$)			
	Baseline	Fast	Treatment	Change	Baseline	Fast	Treatment	Change	Baseline	Fast	Treatment	Change
FIE (*deg*)	15.18 (2.61)	18.93 (4.90)	20.55 (3.61)	35.81%* (13.01)	16.07 (2.38)	20.79 (2.87)	21.58 (1.98)	36.23%* (19.80)	16.26 (2.93)	19.63 (3.43)	19.90 (2.38)	23.13%* (11.95)
Stride Length (*m*)	1.34 (0.15)	1.51 (0.20)	1.55 (0.21)	15.04%* (8.94)	1.34 (0.14)	1.55 (0.12)	1.53 (0.13)	14.04%* (9.19)	1.32 (0.12)	1.47 (0.13)	1.51 (0.14)	14.45%* (5.50)
Cadence (step/min)	109.47 (9.17)	131.44 (12.60)	113.06 (11.22)	3.37% (7.05)	112.66 (6.95)	131.81 (7.81)	112.75 (11.19)	0.09% (8.54)	104.47 (11.85)	118.74 (0.21)	111.08 (10.10)	6.81%* (7.66)
Speed (*m/s*)	1.22 (0.18)	1.65 (0.27)	1.46 (0.26)	18.90%* (12.43)	1.26 (0.15)	1.70 (0.19)	1.44 (0.20)	14.32%* (15.19)	1.15 (0.20)	1.45 (9.69)	1.40 (0.22)	22.43%* (13.08)
Reaction time (*ms*)	593 (151)	-	620 (147)	5.22%* (7.09)	623 (199)	-	697 (232)	12.09%* (12.19)	634 (220)	-	638 (243)	0.53% (10.75)

With (*) Showing Percent Changes Significantly Different From **0%**
